# Delayed Onset Postoperative Spinal Epidural Hematoma after Lumbar Spinal Surgery: Incidence, Risk Factors, and Clinical Outcomes

**DOI:** 10.1155/2020/8827962

**Published:** 2020-12-23

**Authors:** Longjie Wang, Hui Wang, Yan Zeng, Woquan Zhong, Zhongqiang Chen, Weishi Li

**Affiliations:** Department of Orthopaedic, Peking University Third Hospital, Beijing, China

## Abstract

**Background:**

Posterior spinal epidural haematoma (PSEH) often develops within 24 hours after surgery. On rare occasions, PSEH occurs after 3 days and up to two weeks and is classified as delayed-onset PSEH. Due to its rarity, previous studies have only described the clinical features, whereas risk factors have not been assessed.

**Methods:**

Patients who developed PSEH requiring haematoma evacuation between December 2013 and January 2020 were included and divided into the early-onset (group A) and delayed-onset (group B) groups based on the time of symptom onset (>72 hours). For each PSEH patient, 3 controls (group C) who did not develop PSEH in the same period were randomly selected. Clinical features were compared among the three groups, and multiple logistic regression analysis was performed to identify the risk factors for groups A and B.

**Results:**

Thirty-two patients (0.35%) were identified as having early-onset PSEH (occurring at 10.68 ± 11.5 h), and 15 (0.16%) patients had delayed-onset PSEH (occurring at 130.60 ± 61.78 h). When comparing groups A and B, group A showed a higher rate of multilevel procedures, lower drainage, lower APTT, and higher JOA score at discharge. Multiple logistic regression analysis identified multilevel procedures (OR: 5.62, 95% CI: 1.84-17.25), postoperative systolic blood pressure (SBP) (OR: 1.10, 95% CI: 1.06-1.15), and abnormal coagulation (OR: 5.68, 95% CI: 1.74-18.52) as independent risk factors for group A, whereas postoperative SBP (OR: 1.10, 95% CI: 1.04-1.16) and previous spinal surgery (OR: 4.74, 95% CI: 1.09-20.70) at the same level were risk factors for group B.

**Conclusions:**

Our study revealed that the overall incidence of delayed-onset PSEH was 0.16% in posterior lumbar spinal surgery and that its risk was different from that of early-onset PSEH. If patients with such risk factors develop neurological deficits 3 days after initial surgery, surgeons should be aware of the possibility of delayed-onset PSEH.

## 1. Introduction

Postoperative spinal epidural haematoma (PSEH) is a rare complication after spinal surgery but can lead to devastating neurological deficits, including sensory disturbance, lower extremity weakness, paralysis, and bowel dysfunction [1, 2]. Since Jackson first described it as a clinical diagnosis in 1869, more than 1000 cases have been reported in the literature with incidence, aetiology, risk factors, and outcomes. The incidence of PSEH is 0.1%-0.4% and is increased in patients with age > 60, high body mass index (BMI), previous spinal surgery, coagulopathy, use of nonsteroidal drugs, intraoperative blood loss > 1 L, use of gelfoam, and multilevel procedures [3–7].

In clinical practice, PSEH often develops shortly after surgery, especially in 24 hours. However, on rare occasions, PSEH occurs after 3 days and up to two weeks and is classified as delayed-onset PSEH [8]. This uncommon clinical entity usually leads to misdiagnosis because of atypical clinical features, resulting in delayed operation and worse outcomes. The cause of delayed-onset PSEH is still unknown. Although multiple studies have suggested surgeons keep in mind delayed-onset PSEH after surgery, due to its rarity, previous studies have only described the clinical features based on case reports or a small sample cohort, and risk factors have not been assessed. For example, Spanier DE et al. first described an epidural haematoma that occurred 16 days after index surgery in an elderly patient who received heparin therapy [9]. Uribe Juan et al. reported 7 cases of PSEH occurring with an average time of 5.7 days after original surgery over a 4-year period and observed a high ratio of previous spinal surgery in such patients [8]. Masato Anno et al. enrolled 14 PSEH patients and found that almost half of the cases occurred during the delayed phase, drawing the conclusion that patients with spinal surgeries should be followed up carefully for approximately one week.

Therefore, we aimed to investigate the incidence and clinical features of delayed-onset PSEH and identify its risk factors based on patients who underwent posterior lumbar spinal surgery with instrumentation to determine if any common elements predispose them to development. Moreover, we enrolled early-onset PSEH and compared the risk factors, clinical features, and outcomes between the two types of PSEH.

## 2. Materials and Methods

### 2.1. Subjects

This study was approved by the ethics board of our hospital; for this type of study, informed consent was waived. Patients who underwent posterior lumbar decompression surgery for lumbar spinal stenosis caused by degenerative disease and disc herniation with instrumentation in our hospital between December 2013 and January 2020 were included in this study. The PSEH group patients were diagnosed by symptoms (back pain at the surgical level, radicular pain at lower limbs, and/or neurological deterioration), radiological examination (MRI and/or CT), and intraoperative findings (haematoma compressing the dura) and underwent haematoma evacuation. The PSEH group was divided into early-onset (group A) and delayed-onset groups (group B) based on the time of symptom onset (>72 hours). For the control group (group C), those who did not develop PSEH in the same period were randomly selected using a random number table from the pool of patients. All patients underwent surgery under general anaesthesia, and drainage tubes were routinely placed during the surgery. The drainage tube was withdrawn when the volume was less than 50 mL every 24 hours. The exclusion criteria were as follows: (1) LSS caused by trauma, tumour, infection, deformity, or other diseases; (2) PSEH occurring at the thoracolumbar junction; (3) initial surgeries performed at other hospitals; and (4) incomplete clinical data.

### 2.2. Data Collection

Clinical data for PSEH, including preoperative factors, perioperative factors, postoperative factors, laboratory data, and blood pressure at different times, were reviewed.

Preoperative factors, including age, sex, body mass index (BMI), comorbidities, previous spinal surgery at the same level, smoking and drinking status, recent medication record (anticoagulation and nonsteroidal drug), course of disease, and radiographic parameters (lumbar lordosis and local lordosis angle), were recorded. The comorbidities included hypertension, coronary disease, cerebral infarction, and diabetes, which may have had an impact on clinical outcomes. Radiographic parameters were recorded from full-length spine lateral X-rays using Surgimap software (Nemaris, USA) [10, 11]. Lumbar lordosis was measured from the upper endplate of L1 to the upper endplate of S1. The local lordosis angle was measured from the upper endplate of the upper diseased vertebrae to the lower endplate of the lower diseased vertebrae or the upper endplate of S1. Perioperative factors, including the multilevel procedure, length of surgery, estimated blood loss, dural tear, gelfoam dural coverage, and number of drainage tubes, were recorded. A multilevel procedure was defined as a surgical procedure involving more than one intervertebral disc. Postoperative factors, including an increase in blood pressure after extubation, a >50 mmHg increase in blood pressure after extubation and drainage at evacuation surgery, were recorded. The increase in blood pressure after extubation was calculated as the difference between the average intraoperative blood pressure and highest blood pressure after extubation before returning to wards.

Laboratory data were collected, including routine blood tests (blood cell number, haemoglobin level, haematocrit, and platelet count) and coagulation status (prothrombin time, activated partial thromboplastin time, thrombin time, fibrinogen, and international standardization rate). Abnormal coagulation was evaluated by platelet count, prothrombin time, and activated partial thromboplastin time according to our laboratory standards.

Blood pressure was measured at admission, intraoperatively, after extubation and postoperatively. The intraoperative blood pressure was measured every 10 minutes following initiation of general anaesthesia and was recorded as an average level.

### 2.3. Literature Review

We also conducted a brief review of published articles that reported delayed-onset PSEH between 1998 and 2019 using PubMed. The database search was limited to publications enrolled in delayed-onset PSEH in English. The keywords were “spinal epidural haematoma” and “delayed-onset spinal epidural haematoma”.

### 2.4. Statistical Analysis

The results were recorded and analysed by SPSS software (version 24, IBM, Armonk, New York). Data are presented as the means ± standard deviation. Between each two groups, continuous data were assessed by independent sample *t* test, and categorical data were assessed by *χ*^2^ test and Fisher's exact test. Univariate analysis was first carried out to identify the potential risk factors. Then, multiple logistic regression analysis was applied to identify the independent risk factors for each type of PSEH, and adjusted odds ratios with 95% confidence intervals were calculated. According to the results of the univariate analysis, factors with a *P* value less than 0.05 and significant clinical importance (whether *P* value was less than 0.05 or not) were selected for the multiple logistic regression. A *P* value < 0.05 was considered significant.

## 3. Results

From December 2013 to January 2020, 9258 patients underwent posterior lumbar decompression surgery for lumbar spinal stenosis at our hospital. Forty-seven patients (0.51%) were diagnosed with PSEH after the initial surgery and underwent haematoma evacuation. Among them, 32 patients (68.09%) were identified as having early-onset PSEH (occurring at 10.68 ± 11.5 h after initial surgery), and 15 patients (31.91%) had delayed-onset PSEH (occurring at 130.60 ± 61.78 h after initial surgery). Another 141 patients who did not develop PSEH were enrolled as the control group.

### 3.1. Univariate Analysis

For preoperative factors, there was no significant difference in the ratio of males to females, age, comorbidities, smoking and drinking status, recent medication record, and course of the disease or local lordosis angle between each two groups. When compared with group C, group A showed a higher BMI (26.31 ± 2.99 kg/m^2^), whereas group B showed a higher rate of previous spinal surgery at the same level (26.67%) and larger lumbar lordosis (25.95 ± 27.99°). For perioperative factors, estimated blood loss (395.00 ± 193.71 mL vs. 506.67 ± 419.56 mL vs. 446.92 ± 324.44 mL), duration of surgery (152.20 ± 35.53 min vs. 159.64 ± 52.70 min vs. 144.59 ± 44.46 min), dural tear, and gelfoam dural coverage showed no significant differences among groups A, B, and C. Only the rate of the multilevel procedure was significantly higher in group A than in groups B and C. For postoperative factors, the increase in systolic blood pressure (SBP) in group C was significantly lower than those in the two types of PSEH. The drainage in group A was significantly lower than that in groups B and C. These data are listed in Table 1.

Laboratory data are listed in Table 2. Only APTT and postoperative haemoglobin showed significant differences among groups A, B, and C. There was no significant difference in other laboratory data collected in this study among the three groups. According to the coagulation status, 11 patients in group A, 4 patients in group B, and 20 patients in group C were classified as having abnormal coagulation.

The comparison of clinical presentation between groups A and B is shown in Table 3. The times from onset to evacuation were 24.72 ± 38.79 h in group A and 63.40 ± 57.87 h in group B. The symptoms in group A were dysesthesia in 15 patients, pain in 5 patients, and muscle weakness in 29 patients, and the symptoms in group B were dysesthesia in 5 patients, pain in 1 patient, and muscle weakness in 15 patients. The JOA scores were similar in the two PSEH groups prior to surgery but were significantly worse in the delayed-onset PSEH group than in the early-onset group at discharge (group A: 20.68 ± 4.72, group B: 17.67 ± 4.79, *P* = 0.039, <0.05).

Blood pressure measured at different time points is listed in Table 4. In the control group, SBP at admission, after extubation and postoperatively, was significantly lower than those in the two types of PSEH, whereas DBP after extubation was significantly lower than that in group A.

### 3.2. Multiple Logistic Regression Analysis (Table 5)

For group A, multiple logistic regression analysis identified multilevel procedures (adjusted odds ratio: 5.62, 95% confidence interval: 1.84-17.25), postoperative SBP (adjusted odds ratio: 1.10, 95% confidence interval: 1.06-1.15), and abnormal coagulation (adjusted odds ratio: 5.68, 95% confidence interval: 1.74-18.52) as independent risk factors.

For group B, multiple logistic regression analysis identified postoperative SBP (adjusted odds ratio: 1.10, 95% confidence interval: 1.04-1.16) and previous spinal surgery at the same level (adjusted odds ratio: 4.74, 95% confidence interval: 1.09-20.70) as independent risk factors.

### 3.3. Literature Review (Table 6)

Twenty-two patients with delayed-onset PSEH were reported in the literature during the past 21 years [8, 9, 12–17]. Among them, 5 studies were case reports, and the other 3 were case series studies. Only 2 studies reported the incidence and recovery rate. Detailed information and speculated contributing factors are listed in Table 6.

## 4. Discussion

In the present study, over a 7-year period, 47 of 9258 patients (0.51%) developed PSEH after surgery; among them, 31.91% had delayed-onset haematoma (0.16%). Postoperative SBP and previous spinal surgery at the same level were identified as independent risk factors for its development. Furthermore, early- and delayed-onset PSEH had different risk factors. To the best of our knowledge, this is the first study to reveal the incidence of delayed-onset PSEH in patients undergoing posterior lumbar spinal surgery and its risk factors.

### 4.1. Incidence

Multiple studies have described delayed-onset PSEH in patients undergoing all levels of spinal surgery [8, 13, 15–17]. However, because of its rarity, most studies have been case reports and did not obtain the incidence. Based on our review, only three articles enrolled more than one case in their studies, and two of them estimated the incidence. Uribe J et al. enrolled 7 patients and first defined delayed PSEH as an uncommon cause of delayed neurological deterioration, which took more than three days and up to two weeks after the initial surgery. They reported an incidence of 0.17%, whereas Anno Masato enrolled 6 patients and reported an incidence of 0.18% [8, 17]. In our practice, to avoid the influence of anatomical characteristics of different regions, we limited our study to cases with posterior lumbar spinal surgery. Fifteen patients who enrolled in the study were asymptomatic in the first 3 days after initial surgery and then developed neurological deterioration for an average duration of 134.6 hours. The prevalence was 0.16%, which was close to the previous two studies, indicating that the incidence is similar regardless of the region.

### 4.2. Risk Factors

The relationship between blood pressure and haematoma has been widely studied in previous studies [4, 5, 18, 19]. According to our study, although univariate analysis revealed that SBP at different time points except for intraoperative SBP was significantly higher in the delayed-onset PSEH group, further multiple logistic regression analysis revealed that only patients with high postoperative SBP had a 1.10-fold increased risk of developing haematoma. Two possible explanations may account for this finding. First, patients usually present an elevation in blood pressure after surgery because of wound pain, but the self-regulation mechanisms can rapidly adjust the vessel size to decrease blood pressure. However, in these poorly managed or undiscovered hypertension patients, owing to hardening vessels, the self-regulation mechanism fails to regulate vessel size, and high blood pressure may lead to haemorrhage again after surgery [18, 20]. Second, previous studies have reported a positive relationship between whole blood viscosity (WBV) and hypertension [21–23]. The high postoperative SBP may result in a high WBV that could cause blood clots and block the drainage tube. Therefore, both preoperative hypertension and postoperative hypertension need to be controlled immediately, consistent with previous studies. Ohba Tetsuro et al. found that, among PSEH patients, 4 late-onset patients had obvious postoperative hypertension [20]. Another study from Japan showed that 83.3% of PSEH patients were in a hypertensive state after surgery, and the development of PSEH could be prevented by controlling blood pressure.

Another risk factor independently associated with delayed-onset PSEH is previous spinal surgery at the same level. Many previous studies have investigated the role of this factor in PSEH [1, 4, 19]. However, only one study identified it as a risk factor [19]. Moreover, a study on delayed-onset PSEH revealed that the rate of previous spinal surgery was much higher than that in the control group [8]. However, the samples were too small to conduct a statistical analysis. In our study, based on the information collected, we drew a similar conclusion as these two studies. It is possible that the second surgery is more difficult and traumatic due to scar tissue, increasing the risk of bleeding.

Interestingly, in our study, we first found that the risk factors were different for the two types of PSEH, indicating that their mechanisms were different. This may be one reason why previous studies obtained controversial results. The proportion of delayed-onset PSEH in enrolled samples could influence the statistical analysis.

### 4.3. Clinical Features

With regard to initial symptoms, our study was consistent with previous findings that lumbar PSEH often leads to paralysis and pain [1, 17]. In our study, muscle weakness was the most common symptom regardless of the time of onset, following dysesthesia and pain. By comparing the two types of PSEH, although the JOA scores were similar in the two PSEH groups prior to surgery, they were significantly worse in the delayed-onset PSEH group than in the early-onset group at discharge. Immediate surgical intervention seems to influence this. It is believed that patients with short symptom durations and rapid surgical intervention are correlated with better outcomes [24, 25]. However, the time from onset to evacuation in delayed-onset PSEH is much longer than that in early-onset PSEH, indicating that spinal surgeons may ignore the possibility of PSEH occurring after 3 days and treat it as nerve oedema.

This study had several limitations. First, because of the retrospective nature of the study, missing data, bias and confounders were inevitable, which may have affected the outcomes. Second, due to the rarity of delayed-onset PSEH, the sample size was relatively small, and a prospective study could not be conducted to verify the risk factors. Future multicentre studies are necessary to solve this problem. Third, because the spinal epidural plexus vein system is a valveless network, a sudden increase in abdominal pressure, such as a bad cough and sneezing, may lead to rupture [26]. Similar causes may have affected the results.

## 5. Conclusions

In conclusion, our study revealed that, among patients with PSEH, 31.91% had delayed onset, and the overall incidence was 0.16% in posterior lumbar spinal surgery. Further study found that postoperative SBP and previous spinal surgery at the same level were independent risk factors. We also found that the risk factors for early- and delayed-onset PSEH were different. Our results remind surgeons to be aware of the possibility of delayed-onset PSEH; if patients with such risk factors develop neurological deficits after 3 days, surgeons should make a timely diagnosis.

## Figures and Tables

**Table 1:** Comparison of preoperative, perioperative, and postoperative factors among three groups.

	Group A (early onset)	Group B (delayed onset)	Group C (control group)	*P*_A−C_	*P*_B−C_	*P*_A−B_
Preoperative factors						
Age (years)	56.65 ± 7.75	52.73 ± 13.84	55.72 ± 14.34	0.72	0.44	0.22
Sex (male/female)	15/17	8/7	88/53	0.11	0.49	0.68
BMI(kg/m^2^)	26.31 ± 2.99	25.00 ± 3.10	24.32 ± 2.47	**<0.05**	0.32	0.18
Anticoagulation	4 (12.50%)	1 (6.67%)	6 (4.26%)	0.09	0.51	1.00
Nonsteroidal drug	18 (56.25%)	10 (66.67%)	88 (62.41%)	0.52	0.50	0.27
Hypertension	17 (53.13%)	6 (40%)	50 (35.46%)	0.064	0.73	0.40
Hypertension treatment	10 (31.25%)	3 (20%)	49 (34.75%)	0.71	0.25	0.42
Coronary disease	8 (25.00%)	2 (13.33%)	27 (19.14%)	0.47	0.74	0.47
Cerebral infarction	3 (9.38%)	1 (6.67%)	5 (3.55%)	0.17	0.46	1.00
Diabetes (%)	6 (18.75%)	1 (6.67%)	13 (9.22%)	0.13	0.74	0.28
Smoking or drinking	2	0	12	1.00	0.61	1.00
Previous spinal surgery at the same level (%)	4 (12.50%)	4 (26.67%)	8 (5.67%)	0.24	**0.018**	0.25
Course of the disease (years)	9.64 ± 8.74	7.91 ± 7.97	9.58 ± 6.41	0.96	0.36	0.53
Lumbar lordosis (°)	33.37 ± 24.88	25.95 ± 27.99	34.76 ± 13.97	0.63	**0.042**	0.18
Local lordosis angle (°)	21.06 ± 12.39	19.60 ± 15.58	20.90 ± 6.27	0.92	0.55	0.75
Perioperative factors						
Multilevel procedure (%)	24 (75.00%)	6 (40.00%)	56 (39.72%)	**<0.05**	0.98	**0.02**
Estimated blood loss (mL)	395.00 ± 193.71	506.67 ± 419.56	446.92 ± 324.44	0.39	0.51	0.22
Duration of surgery (min)	152.20 ± 35.53	159.64 ± 52.70	144.59 ± 44.46	0.37	0.24	0.58
Dural tear (%)	4 (12.5%)	1 (6.67%)	8 (5.67%)	0.25	1.00	1.00
Gelfoam dural coverage (%)	21 (65.63%)	11 (73.33%)	72 (51.06%)	0.14	0.10	0.74
Number of drainage tube (number)	1.09 ± 0.29	1.07 ± 0.26	1.10 ± 0.30	0.93	0.69	0.76
Postoperative factors						
Increase of SBP after extubation (mmHg)	27.50 ± 16.11	30.00 ± 18.29	20.88 ± 13.22	**0.015**	**0.019**	0.64
Increase of DBP after extubation (mmHg)	23.23 ± 13.76	20.71 ± 13.84	18.11 ± 7.85	**0.005**	0.28	0.57
≥50 mmHg increase in SBP after extubation (%)	6 (18.8%)	2 (13.3%)	15 (10.6%)	0.23	0.67	1.00
Drainage (mL)	239.69 ± 245.70	613.67 ± 599.83	688.19 ± 295.81	**<0.05**	0.41	**0.004**

Values in bold indicates *P* value < 0.05, which is considered as significance difference. BMI: body mass index. Multilevel procedure was defined as a surgical procedure involved more than one intervertebral disc. Increase of blood pressure after extubation was calculated as difference between average intraoperative blood pressure and highest blood pressure after extubation before back to wards.

**Table 2:** Laboratory data.

	Group A (early onset)	Group B (delayed onset)	Group C (control group)	*P*_A−C_	*P*_B−C_	*P*_A−B_
Before surgery						
Red blood cell (×10^9^/L)	4.64 ± 0.49	4.63 ± 0.62	4.80 ± 0.58	0.16	0.31	0.96
Hb (g/L)	140.71 ± 18.87	144.21 ± 17.30	146.70 ± 18.61	0.11	0.63	0.56
HCT	0.42 ± 0.05	0.43 ± 0.05	0.41 ± 0.03	0.38	0.075	0.51
PLT (×10^9^/L)	237.90 ± 106.02	237.93 ± 69.72	227.09 ± 55.68	0.42	0.49	0.99
PT (s)	10.45 ± 0.69	10.65 ± 0.98	10.54 ± 1.11	0.66	0.72	0.43
APTT (s)	31.55 ± 4.07	35.63 ± 7.24	34.91 ± 4.21	**<0.05**	0.56	**0.018**
TT (s)	14.35 ± 1.38	13.63 ± 1.62	14.46 ± 1.58	0.72	0.06	0.13
Fib (g/L)	2.89 ± 0.65	2.96 ± 0.61	2.96 ± 0.56	0.49	0.97	0.71
INR	0.98 ± 0.06	0.99 ± 0.08	1.01 ± 0.14	0.24	0.67	0.51
Abnormal coagulation (%)	11 (34.35%)	4 (26.7%)	20 (14.18%)	**0.007**	0.25	0.74
After surgery						
Red blood cell (×10^9^/L)	3.52 ± 0.45	3.75 ± 0.45	3.70 ± 0.47	0.07	0.67	0.11
Hb (g/L)	105.33 ± 16.68	115.72 ± 40.35	113.88 ± 5.98	**<0.05**	0.62	0.28
HCT	0.32 ± 0.044	0.36 ± 0.032	0.35 ± 0.042	0.001	0.35	0.005
PLT (×10^9^/L)	207.64 ± 210.72	209.38 ± 73.71	210.71 ± 50.44	0.81	0.93	0.95

Values in bold indicates *P* value < 0.05, which is considered as significance difference. Hb: hemoglobin; HCT: hematocrit; PLT: platelet count; PT: prothrombin time; APTT: activated partial thromboplastin time; TT: thrombin time; Fib: fibrinogen; INR: international normalized ratio. Abnormal coagulation was evaluated by platelet count, prothrombin time, and activated partial thromboplastin time according to our laboratory standards.

**Table 3:** Clinical presentation.

	Group A (early onset)	Group B (delayed onset)	*P*_A−B_
Onset (h)	10.68 ± 11.51	130.60 ± 61.78	**<0.05**
Time from onset to evacuation (h)	24.72 ± 38.79	63.40 ± 57.87	**0.009**
Symptoms			
Dysesthesias (%)	15 (30.6%)	5 (23.8%)	0.58
Pain (%)	5 (10.2%)	1 (4.8%)	
Muscle weakness (%)	29 (59.2%)	15 (71.4%)	
JOA score			
Admission	13.44 ± 5.39	11.67 ± 5.54	0.18
Discharge	20.68 ± 4.72	17.67 ± 4.79	**0.039**

Values in bold indicates *P* value < 0.05, which is considered as significance difference.

**Table 4:** Blood pressure at different time point.

	Group A (early onset)	Group B (delayed onset)	Group C (control group)	*P*_A−C_	*P*_B−C_	*P*_A−B_
SBP at admission (mmHg)	138.15 ± 19.45	135.86 ± 19.46	124.38 ± 21.30	**0.001**	**0.047**	0.71
DBP at admission (mmHg)	83.81 ± 11.22	81.80 ± 17.93	79.95 ± 10.80	0.07	0.56	0.64
Intraoperative SBP (mmHg)	123.34 ± 9.11	120.90 ± 10.53	121.35 ± 6.55	0.16	0.82	0.43
Intraoperative DBP (mmHg)	74.08 ± 15.14	75.40 ± 9.62	73.15 ± 6.80	0.60	0.26	0.77
SBP after extubation (mmHg)	148.13 ± 16.10	149.07 ± 16.65	139.95 ± 5.92	**<0.05**	**<0.05**	0.85
DBP after extubation (mmHg)	91.05 ± 13.72	86.00 ± 8.06	88.07 ± 4.58	**0.016**	0.13	0.16
Postoperative SBP (mmHg)	151.75 ± 22.31	144.20 ± 14.24	129.89 ± 12.11	**<0.05**	**<0.05**	0.24
Postoperative DBP (mmHg)	88.13 ± 12.90	83.33 ± 9.02	84.99 ± 9.02	0.11	0.50	0.20

Values in bold indicates *P* value < 0.05, which is considered as significance difference. SBP: systolic blood pressure; DBP: diastolic blood pressure.

**Table 5:** Multiple logistic regression analysis.

	Adjusted OR	95% CI	*P* value
Group A (early onset)			
Multilevel procedure	5.62	1.84-17.25	0.002
Postoperative SBP	1.10	1.06-1.15	<0.05
Abnormal coagulation	5.68	1.74-18.52	0.004
Group B (delayed onset)			
Postoperative SBP	1.10	1.04-1.16	0.001
Previous spinal surgery at the same level	4.74	1.09-20.70	0.038

OR: odds ratio; CI: confidence interval.

**Table 6:** Literature review.

Author and year	Number	Age (years), sex	Incidence	Disease level	Surgical approach	Time to onset (days)	Recovery rate	Speculated contributing factors
Spanier DE et al. 2000	1	80 F	N/A	L3-S1	L3–S1 laminectomy and instrumented bilateral lateral fusion	16	N/A	Heparin therapy and thrombocytopenia
Uribe J et al. 2003	7	62 3F/4M	0.17%	3C, 1CT, 2T, 1L	Laminectomy, Laminoplasty	5.7	71.43%	Previous spinal surgery
Neo Masashi et al. 2006	1	59 M	N/A	C3-T1	C3–T1 laminoplasty	9	N/A	None
Sokolowski MJ et al. 2006	4	73.5 2F/2M	N/A	3L, 1CT	Posterior laminectomy, anterior discectomy	12.5	N/A	Elderly patients underwent multilevel procedures
Parthiban et al. 2008	1	34 M	N/A	T8	T7 to T9 laminectomy	3	N/A	Paraspinal muscle stretching (arterial bleeding)
Kim Boram et.al 2010	1	23 M	N/A	L3	En bloc spondylectomy	9	N/A	Muscular branch of venous plexus
Tomii Masato et al. 2018	1	56 M	N/A	C4-6	Cervical laminoplasty	7	N/A	Hypertension
Anno Masato et al. 2018	6	67.2 N/A	0.18%	1C, 2T, 3L	N/A	5	80%	None

Disease level: C: cervical; T: thoracic; L: lumbar; CT: cervicothoracic.

## Data Availability

The data used and analyzed during the current study was available from the corresponding author on reasonable request.
